# High Prevalence of the Liver Fluke *Amphimerus* sp. in Domestic Cats and Dogs in an Area for Human Amphimeriasis in Ecuador

**DOI:** 10.1371/journal.pntd.0003526

**Published:** 2015-02-03

**Authors:** Manuel Calvopiña, William Cevallos, Richard Atherton, Matthew Saunders, Alexander Small, Hideo Kumazawa, Hiromu Sugiyama

**Affiliations:** 1 Centro de Biomedicina, Carrera de Medicina, Universidad Central, Quito, Ecuador; 2 Royal Liverpool University Hospital, Liverpool, United Kingdom; 3 Sandwell and West Birmingham Hospitals NHS Trust, Birmingham, United Kingdom; 4 Department of Parasitology Kochi Medical School, Kochi, Japan; 5 Department of Parasitology, National Institute of Infectious Diseases, Tokyo, Japan; Universidad Peruana Cayetano Heredia, PERU

## Abstract

**Background:**

*Amphimerus* sp. is a liver fluke which recently has been shown to have a high prevalence of infection among an indigenous group, Chachi, who reside in a tropical rainforest in the northwestern region of Ecuador. Since it is unknown which animals can act as a reservoir and/or definitive hosts for *Amphimerus* sp. in this endemic area, a study was done to determine the prevalence of infection in domestic cats and dogs. This information is important to understand the epidemiology, life cycle and control of this parasite.

**Methodology/Findings:**

In July 2012, three Chachi communities located on Rio Cayapas, province of Esmeraldas, were surveyed. A total of 89 of the 109 registered households participated in the study. Of the 27 cats and 43 dogs found residing in the communities, stool samples were collected from 14 cats and 31 dogs (total of 45 animals) and examined microscopically for the presence of *Amphimerus* eggs. The prevalence of infection was 71.4% in cats and 38.7% in dogs, with similar rates of infection in all three communities. Significantly more cats were infected than dogs (p = 0.042).

**Conclusions/Significance:**

The data show a high rate of *Amphimerus* sp. infection in domestic cats and dogs residing in Chachi communities. It can be concluded that these animals act as definitive and reservoir hosts for this liver fluke and that amphimeriasis is a zoonotic disease. These findings provide important epidemiological data which will aid in the development and implementation of control strategies against the transmission of *Amphimerus*.

## Author Summary

*Amphimerus* sp. is a fluke that infects the bile ducts of its definitive hosts. Recently, it has been shown that an indigenous Amerindian group, the Chachi, living in a rural and remote tropical area of Ecuador, are infected with this parasite. The epidemiology and life cycle of this parasite remains elusive, and research is needed to understand the mode of transmission and zoonotic potential of the parasite. It was hypothesized that the domestic animals of the Chachi households may act as definitive and reservoir hosts for *Amphimerus* infection. Hence, the presence and prevalence of infection in these animals residing in communities endemic for human amphimeriasis was investigated. Some 45 animal stool samples were examined microscopically for the presence of *Amphimerus* eggs. The results showed an infection rate of 71.4% in cats and 38.7% in dogs. The data provided evidence that these domestic animals act as both definitive and reservoir hosts for the parasite and that amphimeriasis is a zoonotic disease. The implementation of a mass treatment/control program must target both humans and animals in order to minimize the transmission of this liver fluke.

## Introduction

*Amphimerus* Barker, 1911 is a genus of parasitic liver fluke which are flat helminths (Platyhelminthes) of the Trematoda class and belong to the Opisthorchiidae family. The adult flukes reside within the bile ducts of a definitive host [1]. Infection by liver flukes of this family, which include *Clonorchis sinensis* and *Opisthorchis* spp. can occur through the consumption of raw or undercooked, metacercariae infected freshwater fish [1–5]. Liver fluke infection is one of the more important food-borne diseases worldwide and is considered by the World Health Organization as a neglected tropical disease [6]. Affected individuals with liver flukes of the Opisthorchiidae family can suffer from suppurative cholangitis, cholelithiasis and cholangiocarcinoma [3,5,6].

In the Americas, ten species of *Amphimerus* which infect mammals have been described: *A*. *pseudofelineus*, *A*. *pseudofelineus minutus*, *A*. *caudalitestis*, *A*. *price*, *A*. *lancea*, *A*. *parciovatus*, *A*. *bragai*, *A*. *minimus*, *A*. *neotropicalis* and *A*. *ruparupa* [7,8]. In Ecuador, flukes found in the bile ducts of dogs were previously described as *Opisthorchis guayaquilensis* [9] but later, this species, without any further comparative studies was named as being *Amphimerus guayaquilensis*. However, Artigas and Perez (1964) considered to *A*. *guayaquilensis* to be synonym of *A*. *pseudofelineus*. A few years later, *A*. *guayaquilensis* was considered to be distinct from *A*. *pseudofelineus* and was instead regarded as a synonym of *A*. *parciovatus* [7]. Furthermore, Thatcher (1970) did not agree with this synonymy and contemplated *A*. *guayaquilensis* distinct from *A*. *pseudofelineus* because of the extent of the vitellaria. The validity of some species in this genus is controversial, since speciation is based only on morphological and morphometric features present in the adult flukes and the assignment of species names must be regarded as speculative.

*Amphimerus* spp. have been demonstrated to infect birds, reptiles and certain mammals including cats, dogs, ducks, the double-crested cormorant, Amazonian dolphins, opossums (*Didelphis marsupialis*, *Philander opossum*) and the rodent *Nectomys squamipes* [1,9–16]. For other members of the Opisthorchiidae family known to infect humans e.g. *C*. *sinensis* and *Opisthorchis* spp., cats and dogs are the most important animal reservoirs for human infection [2,3,17,18]. However, it is currently unknown whether domestic animals may act as a definitive and/or reservoir host for human transmission in the recently reported focus of infection in Ecuador [10]. Given the similarities between *C*. *sinensis*, *Opisthorchis* spp. and *Amphimerus* spp., it was hypothesized that these mammals may also act as reservoirs for *Amphimerus* sp. infection in Ecuador.

The indigenous group, Chachi, who live along the Rio Cayapas and its tributaries in the north-western coastal rainforest of Ecuador, have been shown to have a high prevalence of infection (15.5% to 34.1%) with *Amphimerus* sp. [10]. Afro-Ecuadorian and mestizo populations living in separate communities but along the same rivers were not found to be infected [10]. The solo infection of the Chachi population was postulated to be related to their cultural tradition of eating smoked fish and food sharing [10,11].

In a previous pilot study, conducted by the authors, in the same endemic communities for human infection, both cats and dogs were found to be positive for *Amphimerus* eggs. There was no evidence of infection in any other animals investigated (e.g. pigs and chickens). The objective of this study was, therefore, to investigate the prevalence of infection of *Amphimerus* sp. in domestic cats and dogs and to determine their role in the transmission in the Ecuadorian villages endemic for human infection.

## Materials and Methods

### Study area

**Fig 1:**
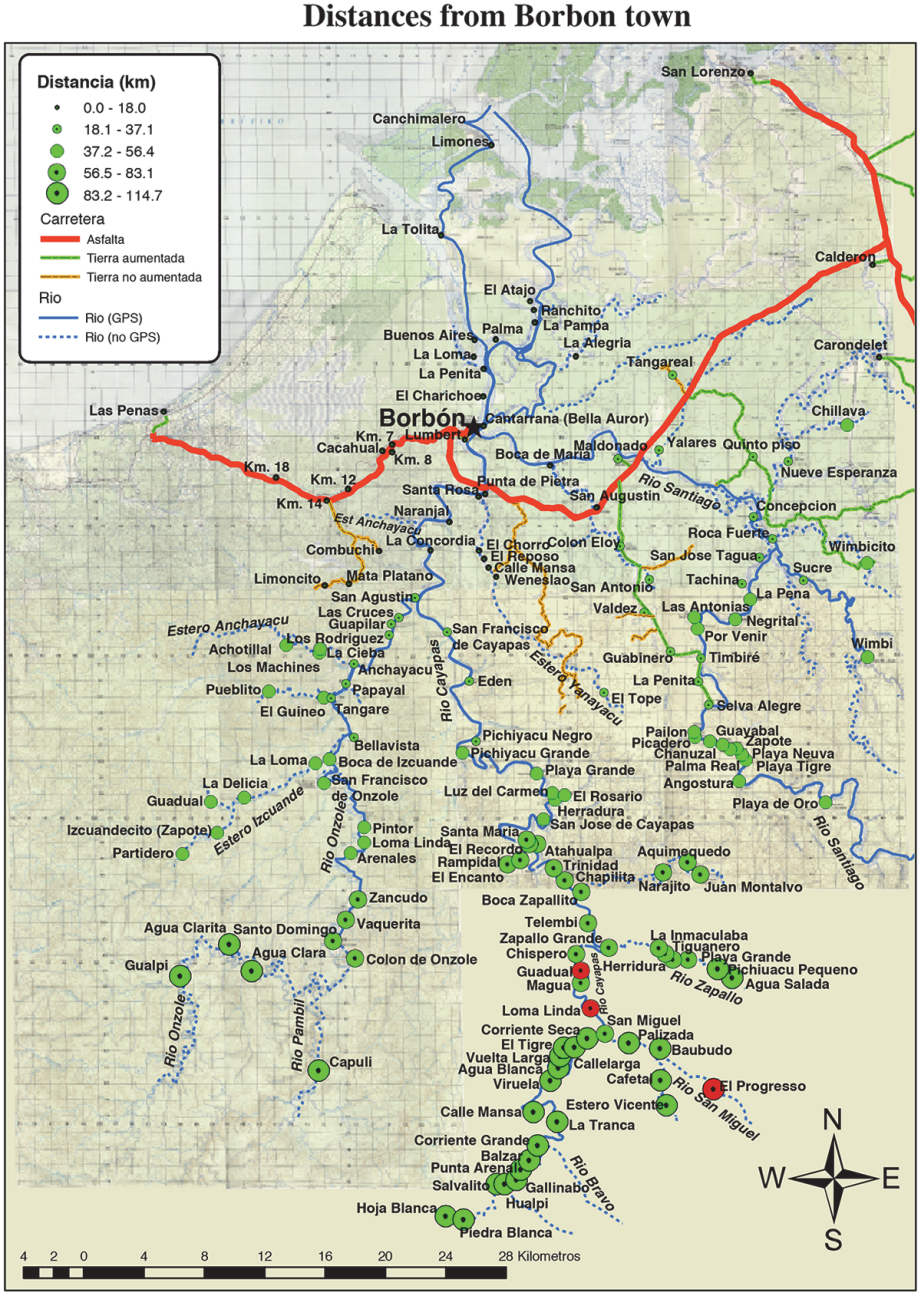
Map of the study area. It showed the geographical location of the study area in the Canton Eloy Alfaro, province of Esmeraldas, 320 km from the capital Quito. In red circles are the 3 communities studied along the Rio Cayapas and its tributary Rio San Miguel.

The study was conducted in three indigenous Chachi communities along the Rio Cayapas in the Province of Esmeraldas, located in the northwest coastal rainforest of Ecuador (Fig. 1). The indigenous Chachi, living alongside Afro-ecuadorian and mestizo populations, is the predominant ethnic group in this area, and represent 13% of the inhabitants in this region of Ecuador [19,20]. These communities are the same as those studied previously, showing the resident Chachi having a high prevalence of infection with *Amphimerus* sp. [10]. They live in remote villages where the only way to reach the communities are by boat along the river; the source of their water is mainly from rivers and streams and is consumed untreated. Sanitation facilities are lacking, and flush toilets are uncommon. The people are hunters and eat fish caught in the neighboring rivers almost every day and the meal is accompanied with cooked rice and plantain.

The province of Esmeraldas, forms part of the tropical rainforest known as “Choco Biogeográfico del Pacífico” which covers a section of the coast of Ecuador, Colombia and Panamá. This area has been labelled as a biological hotspot; an area with an extraordinary concentration of animal species [21]. The climate of this region is warm and humid, with an average temperature of between 24°C and 28°C and an average relative humidity of 85% [22].

### Sampling

This study was based on a previous census conducted in January 2012 where each household was given an identification number and a total of 109 households were recorded in the three communities. In July 2012, all house owners were asked to participate in the study by providing a stool sample from any cats and/or dogs residing in the respective household, simultaneously a census of dogs and cats residing in the participating communities was conducted. A team of community health workers informed the villagers of the study in their local Chapalache language and residents were free to refuse entry to their household or access to their domestic animals at any point during data collection.

### Sample processing

**Fig 2:**
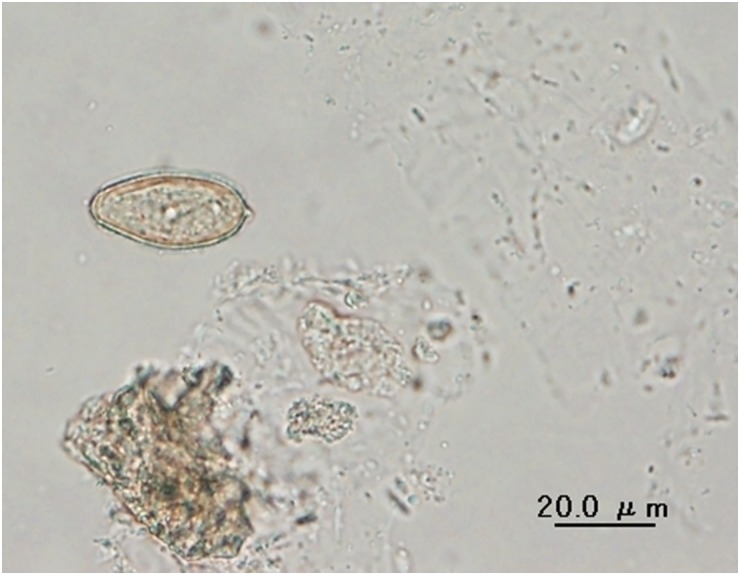
An egg of *Amphimerus* sp. observed in stools from a cat. It is seen to be morphologically similar, using light microscopy, to eggs of *Clonorchis sinensis* and *Opisthorchis* spp. (dimensions 31 μm × 15 μm).

Stool samples were collected by their owners from each animal directly after the deposit was made. Plastic flasks containing stool samples were labelled with type of animal, house code and date. The samples were preserved in 10% formalin and transported to the parasitology laboratory at Centro de Biomedicina in Quito where they were stored at 4°C until they were examined. Samples were concentrated using the formalin-ether technique as previously described [10] and were examined under light microscopy by at least two laboratory technicians for the presence of *Amphimerus* eggs. Samples were then verified by an expert in animal stool examination who was not involved in the data collection. The primary outcome variable was *Amphimerus* infection, defined as positive if eggs of the parasite were visualized by light microscopy. The yellow-brown eggs measured 20 to 32 *u*m in length and 14 to 16 *u*m in width; they are pyriform with a visible operculum at the narrower anterior end. In the centre of the posterior end there is a small bud (Fig. 2).

The adult parasites were obtained from the livers of two cats and one dog. Animals were presented by their owners as sick and showed severe emaciation. They were euthanized with ether and necropsied. The livers were collected in saline solution, squeezed and sliced in small pieces and maintained for 30 minutes. Flukes were then removed from the saline and were fixed in both 70% ethanol and 10% formalin.

### Molecular characterization

For molecular analysis, genomic DNA samples were extracted from each of the *Amphimerus* adult specimens from the cats and dogs using a DNeasy Blood & Tissue Kit (QIAGEN K. K., Tokyo, Japan). The ITS2 region of the ribosomal DNA was then amplified by PCR using Ex Taq DNA polymerase (Takara Bio, Shiga, Japan). The primers used were 3S (forward, 5′-GGTACCGGTGGATCACTCGGCTCGTG-3′) [23] and A28 (reverse, 5′-GGGATCCTGGTTAGTTTCTTTTCCTCCGC-3′) [24]. DNA sequencing of amplicons was performed with a 3100-Advant Genetic Analyzer (Life Technologies, Foster City, CA, USA).

### Statistical analysis

Data was analysed using SPSS version 19 (Statistical Product and Service Solutions, Chicago, IL, USA). The data was stratified by village and animal species, and prevalence of *Amphimerus* infection in the animals calculated for each village. A chi squared test was used to detect any significant differences in the prevalence of infection between animal species and between villages.

### Ethics

Ethical approval of the study was given by ethic committee of the Universidad Central del Ecuador (licence number LEC IORG 0001932, FWA 2482, IRB 2483.COBI-AMPHI-0064–11). All villagers were asked for their verbal consent to access their domestic animals and collect stool samples. Infected animals with any parasite were treated with specific drugs free of charge in the following months. The study was conducted according to the above institution’s guidelines for animal welfare.

## Results

Of the 109 houses recorded in the communities, 89 (81.6%) agreed to take part in the survey (Table 1). From these 89 houses, a total of 27 cats and 43 dogs were counted at the time of data collection. Stool samples from 45/70 animals (64.2%), 14 from cats and 31 from dogs were collected and examined microscopically for the presence of *Amphimerus* eggs (Table 1). Samples of the remaining 25 animals were not able to be collected because they either did not defecate on the collection day or were not present in the home at the time. In cats the prevalence of *Amphimerus* infection was 71.4% (95% confidence interval [CI] = 47.7–95.1), and in dogs, 38.7% (95% CI = 21.6–55.8) (Table 1). The overall prevalence of *Amphimerus* sp. in the two animal species investigated was 48.9% (95% CI = 34.3–63.5). Cats were approximately four times more likely to be infected with *Amphimerus* sp. than dogs, OR = 3.95 (95% CI 1.01–15.6, p = 0.042). There was no statistically significant difference between the communities with regard to prevalence of *Amphimerus* infection in the animals studied.

**Table 1:** Demographics of three villages in northwest Ecuador and the prevalence of *Amphimerus* sp. infection in domestic cats and dogs of these villages.

Village	No. of houses		No. of dogs			No. of cats		
	Total	Surveyed (%)	Total	Examined (%)	Positive (%)	Total	Examined (%)	Positive (%)
1	22	17 (77.2)	10	8 (80)	3 (37.5)	8	4 (50)	3 (75)
2	44	32 (72.7)	19	13 (69)	6 (46.1)	10	5 (50)	3 (60)
3	43	40 (93)	14	10 (72)	3 (30)	9	5 (56)	4 (80)
Total	109	89 (81.6)	43	31 (72)	12 (38.7)	27	14 (52)	10 (71.4)

List of accession numbers/ID numbers for the sequences of the ribosomal DNA ITS2 region of the flukes obtained from humans, cats and dog

Accession numbers, deposited in the GenBank/EMBL/DDBJ nucleotide database are: AB678442 for *Amphimerus* sp. from humans

AB926429 for *Amphimerus* sp. from cats

AB926430 for *Amphimerus* sp. from a dog.

**Fig 3:**
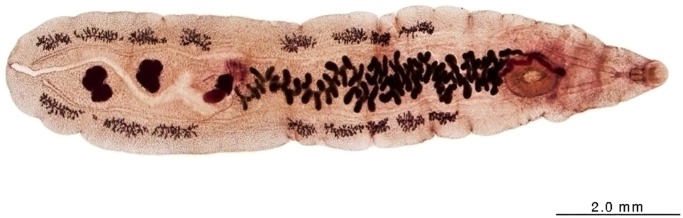
Adult fluke obtained from the biliary ducts of a cat. The major difference of *Amphimerus* flukes from the others Opisthorchiidae is that the vitelline glands situated along both lateral sides of the body are divided into anterior and posterior clusters at the level of the ovary, distributed in four groups, 2 anterior and 2 posterior extending nearly to the excretory pore.

The eggs found in the stools of cats and dogs demonstrated the morphological characteristics consistent with other members of the Opisthorchiidae family (Fig. 2). In order to confirm that the eggs were from *Amphimerus* sp., two positive cats and one positive dog were euthanized and adult flukes were obtained from the bile ducts and subjected to morphological and molecular characterization. The recovered flukes just after extraction from the bile ducts were flat, leaf-like, reddish, flexible and elongated with active movements in saline, with a thinner anterior than posterior extremity, measuring from 15 to 28 mm in length by 2 to 4 mm in width. When fixed in formalin 10%, the flukes became whitish and shorter measuring around 10 to 18 mm long. Flukes were stained with borax carmine and photographed (Fig. 3). Adults of *Amphimerus* sp. can be differentiated from *Clonorchis sinensis* and *Opisthorchis* spp. by certain morphological features e.g.: 1) The presence of two rounded testes, which lie one behind the other in the posterior portion, 2) The vitellaria occupy both lateral sides of the fluke, outside of the intestinal branches and are conspicuously distributed in four groups, 2 anterior and 2 posterior extending backwards nearly to the excretory pore, and 3) the ventral sucker is larger than the oral [1,10]. Furthermore, sequences of the ribosomal DNA ITS2 region of the flukes obtained from the cats and dog were identical to that obtained from humans in the previous study [10]. Accession numbers, deposited in the GenBank/EMBL/DDBJ nucleotide database, are AB678442, AB926429 and AB926430 for *Amphimerus* sp. from humans, cats and dogs, respectively.

## Discussion

The current study is the first to identify *Amphimerus* sp. infection in the domestic animals from Chachi communities in which high rates of infection have been demonstrated [10]. The overall prevalence of *Amphimerus* sp. infection in cats and dogs was relatively high and it can be concluded that these species act as definitive hosts for *Amphimerus* sp. in the study region. As the eggs and adult flukes were demonstrated to be morphologically similar and molecularly identical to those obtained from humans it can be further assumed that they act as reservoir hosts for human infection. This confirms that amphimeriasis is a zoonosis from domestic animals living together with humans. Importantly, the prevalence of infection in the cats and dogs recorded in this study was significantly higher than in the humans of the same communities (48.9% in animals compared to 24% in humans) [10]. These findings suggest that these animals play a role in the transmission of *Amphimerus* to humans. It is of interest to note that the animals studied have contact with villages inhabited by Afro-Ecuadorians and mestizo groups and defecate in and contaminate the community streams, thus represent a risk to these populations which were not found to be infected in the previous human study [10].

The prevalence reported here for *Amphimerus* sp. infection in cats and dogs is similar to that of other members of the Opisthorchiidae family. In China and Korea, the average prevalence of *C*. *sinensis* infection in cats and dogs ranged from 64.1% to 73.2% and from 56.4% to 69.4%, respectively [2]. Furthermore, in a study of *O*. *viverrini* infection in four districts of Thailand, cats had a much higher prevalence (35.5%) than dogs (0.37%) [18].

*Amphimerus* spp. flukes are thought to be transmitted by ingestion of raw or undercooked freshwater fish [1–5]. This was previously shown in 2011, when most of the Chachi included in the study, admitted eating smoked fish caught in nearby rivers [10]. There are two possibilities how these domestic animals can become infected. Firstly, cats and dogs could ingest the leftovers of smoked fish containing the metacercariae of *Amphimerus* sp. Secondly, the animals themselves may swim in the river and feed, thus acquiring infection directly from live fish. The prevalence of *Amphimerus* sp. in cats was significantly higher than in dogs. This is probably because of a cat’s preference for fish and increased capability to catch them in streams. Both cats and dogs have been observed fishing and eating leftovers of human food. Overall 230 freshwater fish from the Cayapas River are currently being examined for metacercariae. A number of trematode metacercariae have been identified but none were *Amphimerus*. It is presumed the intermediate host is only found in the rainy season or the natural infection is very low.

Though the study was successful in identifying a zoonotic link between domestic animals and humans, there are some important limitations that indicate the need for further research in order to validate the findings. Firstly, a total sample size of 45 is small and although this did account for over 64% of all cats and dogs living in the three communities, a larger sample is required to obtain a more accurate estimate of *Amphimerus* infection and more reliable conclusions regarding its transmission dynamics. Furthermore, only one sample was obtained from each animal on one occasion. Although the samples in this study were concentrated to improve sensitivity, future study could involve taking multiple samples on different occasions with duplicate analyses to further improve diagnostic accuracy. On the other hand, surveyed animals were not examined for clinical symptoms, though some were presented sick and emaciated, with others looking healthy. Future research will examine infected animals in more depth and record any visible signs of ill-health.

### Conclusions

This study is of importance in showing that the liver fluke *Amphimerus* sp. can infect and is common in cats and dogs living in Chachi communities of Ecuador, where human amphimeriasis is prevalent. The key finding of the study is that cats and dogs serve as definitive hosts and represent reservoirs for human infection. It can therefore be concluded that amphimeriasis is a zoonotic disease. These results provide relevant data that could be used for policy makers for conducting effective control strategies and measures against *Amphimerus* infection. As this study found a high prevalence of infection in cats and dogs, the recommended public health measure to control transmission would be to treat these domestic animals, as well as humans, with a specific drug for flukes such as praziquantel.
